# Redundant potassium transporter systems guarantee the survival of *Enterococcus faecalis* under stress conditions

**DOI:** 10.3389/fmicb.2023.1117684

**Published:** 2023-02-08

**Authors:** Giuliana Acciarri, Fernán O. Gizzi, Mariano A. Torres Manno, Jörg Stülke, Martín Espariz, Víctor S. Blancato, Christian Magni

**Affiliations:** ^1^Laboratorio de Fisiología y Genética de Bacterias Lácticas, Instituto de Biología Molecular y Celular de Rosario (IBR), Sede Facultad de Ciencias Bioquímicas y Farmacéuticas (FBioyF), Universidad Nacional de Rosario (UNR), Consejo Nacional de Ciencia y Tecnología (CONICET), Rosario, Argentina; ^2^Área Bioinformática, Departamento de Matemática y Estadística, Facultad de Ciencias Bioquímicas y Farmacéuticas, Universidad Nacional de Rosario, Rosario, Santa Fe, Argentina; ^3^Department of General Microbiology, Georg August University, Göttingen, Germany; ^4^Laboratorio de Biotecnología e Inocuidad de los Alimentos, Área de Biotecnología de los Alimentos, FBioyF, UNR–Municipalidad de Granadero Baigorria, Rosario, Argentina

**Keywords:** *Enterococcus faecalis*, potassium transport, KUP/HAK/KT K+ transporters, Ktr family, Kdp system

## Abstract

*Enterococcus* is able to grow in media at pH from 5.0 to 9.0 and a high concentration of NaCl (8%). The ability to respond to these extreme conditions requires the rapid movement of three critical ions: proton (H^+^), sodium (Na^+^), and potassium (K^+^). The activity of the proton F_0_F_1_ ATPase and the sodium Na^+^ V_0_V_1_ type ATPase under acidic or alkaline conditions, respectively, is well established in *these microorganisms*. The potassium uptake transporters KtrI and KtrII were described in *Enterococcus hirae*, which were associated with growth in acidic and alkaline conditions, respectively. In *Enterococcus faecalis*, the presence of the Kdp (potassium ATPase) system was early established. However, the homeostasis of potassium in this microorganism is not completely explored. In this study, we demonstrate that Kup and KimA are high-affinity potassium transporters, and the inactivation of these genes in *E*. *faecalis* JH2-2 (a Kdp laboratory natural deficient strain) had no effect on the growth parameters. However, in KtrA defective strains (Δ*ktrA*, Δ*kup*Δ*ktrA*) an impaired growth was observed under stress conditions, which was restored to wild type levels by external addition of K^+^ ions. Among the multiplicity of potassium transporters identify in the genus *Enterococcus*, Ktr channels (KtrAB and KtrAD), and Kup family symporters (Kup and KimA) are present and may contribute to the particular resistance of these microorganisms to different stress conditions. In addition, we found that the presence of the Kdp system in *E*. *faecalis* is strain-dependent, and this transporter is enriched in strains of clinical origin as compared to environmental, commensal, or food isolates.

## 1. Introduction

*Enterococcus faecalis* is a natural commensal bacterium of the animal gut that can be isolated from diverse niches (water, food, and vegetal; Giraffa, 2002; Byappanahalli et al., 2012). The physiology and genetics of this species are relevant due to its association with infections and diseases, such as bacteremia, endocarditis, urinary tract infections, and dental diseases (Murray, 1990; García-Solache and Rice, 2019). This group of microorganisms emerges as multi-resistant Gram-positive pathogens and, generally, is not regarded as safe (GRAS; Ogier and Serror, 2008). Despite this categorization, *E*. *faecalis* is present in fermented food productions, isolated mainly from traditional dairy products around the world where these bacteria contribute to the ripening process in favorable sensorial features (Giraffa, 2002; Franz et al., 2003). Finally, this microorganism is an important fecal contaminant frequently used as biological indicator for assessing recreational water quality (Boehm and Sassoubre, 2014). Like other members of the lactic acid bacteria, *Enterococcus* has the capability to persist, resist, and thrive in harsh conditions that include low and high pH, heat, and hyperosmotic stress. Members of this genus generally grow at temperatures between 10 and 45°C, in NaCl between 0 and 8% and pH values between 4.5 and 9.0 (Giraffa, 2002; Ogier and Serror, 2008; Byappanahalli et al., 2012).

**Figure 1:**
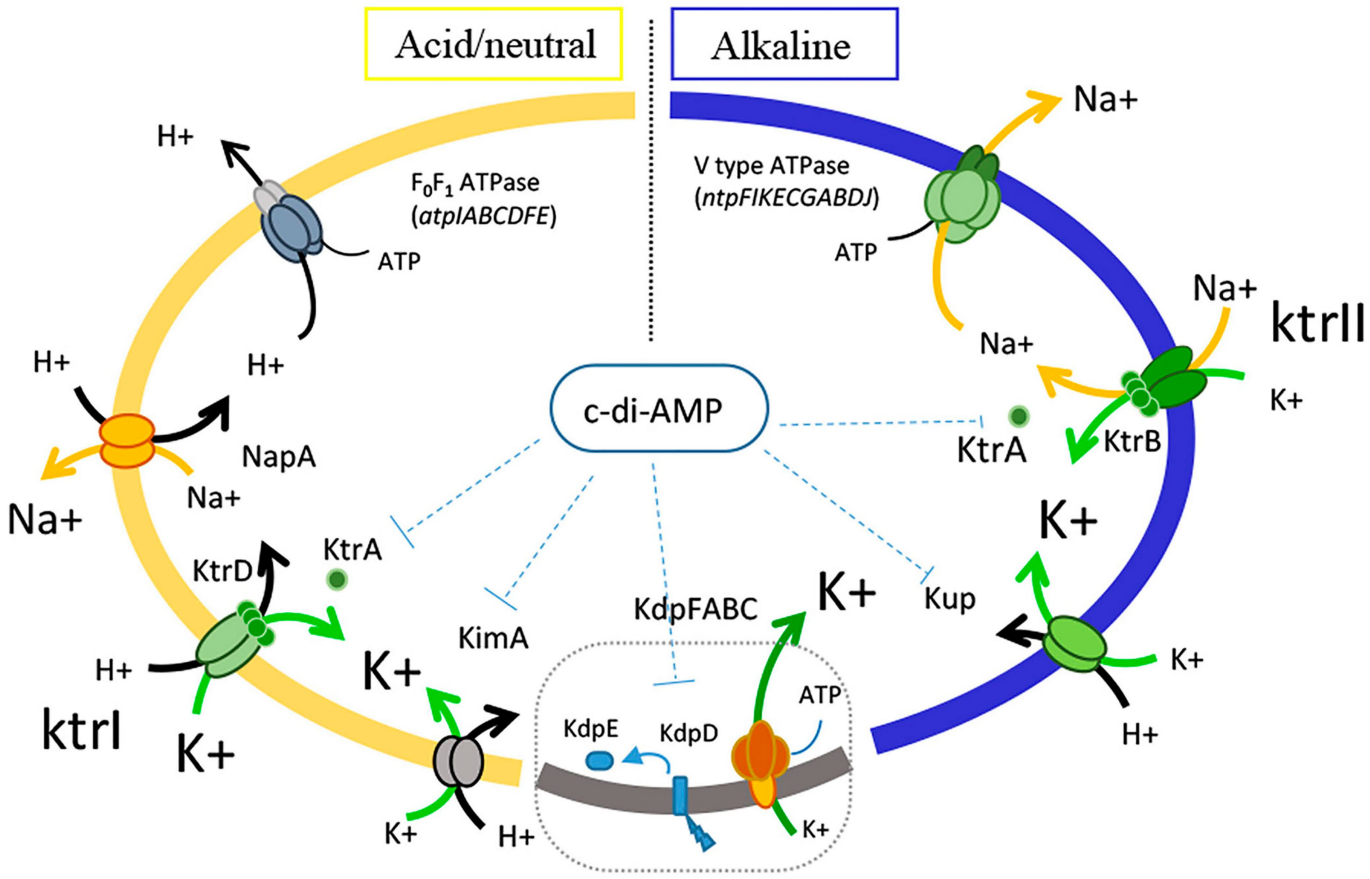
Schematic representation of the mechanisms involved ion fluxes in *Enterococcus faecalis*.

The main biochemistry studies regarding potassium uptake in Enterococci were performed in *Enterococcus hirae* ATCC9790 (Kakinuma, 1998; Krulwich et al., 2011). In this strain, it was well established that the generation of proton-motive force at low pH is generated by proton expulsion *via* the F_0_F_1_ proton translocating ATPase (*atpIABCDFE* operon), extrusion of Na^+^ is catalyzed by NapA antiporter, and the accumulation of cytoplasmic K^+^ catalyzed by KtrI system (Kakinuma, 1998; Krulwich et al., 2011; Figure 1). In alkaline conditions, and in the presence of Na^+^, *E*. *hirae* induces the vacuolar V_0_V_1_ type sodium—ATPase (*ntpFIKECGABDJ* operon) which includes a K^+^ transporter KtrII system (last gene of the operon, *ntpJ*). Thus, Na^+^ is pumped outside the cells consuming ATP whereas K^+^ is accumulated *via* the K^+^/Na^+^ symporter (Kakinuma, 1998; Krulwich et al., 2011; Figure 1).

In the last decade, different K^+^ channels and transporters were characterized in pathogenic and nonpathogenic microorganisms (Stautz et al., 2021). In *Bacillus subtilis*, the KtrAB and KtrCD potassium channels as well as the KimA K+/H+ symporter were characterized (Gundlach et al., 2017). The KtrAB channel and the KimA transporter are high-affinity uptake systems whereas the KtrCD channel has a low affinity unless stimulated by glutamate (Krüger et al., 2020). In *Staphylococcus aureus*, the KdpFABC pump and KtrCB and KtrCD channels are involved in the response to osmotic shock (Gries et al., 2013, 2016; Price-Whelan et al., 2013). In *Streptococcus pneumoniae*, the *trkH cabP* operon encodes a member of the Trk/Ktr/HKT K^+^ channel family (Bai et al., 2014), and in *Streptococcus mutans*, coded by the *trkAH* operon (similar to described in *S*. *pneumoniae*), was defined as the main system involved in the K^+^ uptake in the response to acid stress (Binepal et al., 2016). In *Streptococcus agalactiae*, two members of the Trk/Ktr/HKT family are involved in the uptake of K^+^ (Devaux et al., 2018). In *Streptococcus pyogenes*, the KtrAB system have a main role in K^+^ uptake, while Kup and KimA may have functions under different growth conditions (Faozia et al., 2021). Finally, in *Lactococcus*, two copies of Kup (KupA and KupB) were found in the *Lactococcus lactis* IL1403 strain and only one in the *Lactococcus cremoris* MG1363 (KupB; Pham et al., 2018; Quintana et al., 2019).

The second messenger c-di-AMP was described as involved in the regulation of K^+^ homeostasis in Firmicutes (Corrigan and Gründling, 2013; Stülke and Krüger, 2020; Stautz et al., 2021). Specific interactions between potassium transporters and c-di-AMP were found, c-di-AMP binds to the interface of the KtrA dimer, and inactivates the KtrAB channel (Kim et al., 2015; Rocha et al., 2019). Also, direct interaction of c-di-AMP to KimA (Gibhardt et al., 2019; Gundlach et al., 2019), Kup (Quintana et al., 2019), and CabP (TrkH; Bai et al., 2014; Devaux et al., 2018) was found (Faozia et al., 2021). Additionally, it has been demonstrated that both c-di-AMP and the K^+^ transporters are directly and indirectly involved in the virulence of pathogenic microorganisms (Corrigan and Gründling, 2013; Gries et al., 2013; Stülke and Krüger, 2020; Kundra et al., 2021; Stautz et al., 2021). In *E*. *faecalis* inactivation of the gene encoding for CdaA (*cdaA*, diadenylate cyclase involved in the synthesis of c-di-AMP) or PDE phosphodiesterases (*gdpP* and *dhh*, encoding the enzymes that degrade c-di-AMP) result in the reduction of virulence and capacity to resist osmotic stress (Kundra et al., 2021).

Initial studies of potassium transport systems in *E*. *faecalis* V583 identified the *kdpED* operon, which encodes a two-component regulatory system (KdpD sensor kinase and KdpE transcriptional factor) widely distributed in bacteria, responsible for the transcriptional induction of the KdpFABC transporter complex (potassium-transporting ATPase; Hancock and Perego, 2004). It is also known that the transcriptional induction of *kdp* operon is coordinated with the V type *ntp* ATPase operon in response to the increased salt concentration, demonstrating the importance of the K^+^ uptake and Na^+^ extrusion in the ion homeostasis of the enterococcal cells (Solheim et al., 2014).

The purpose of this study was to study which K^+^ transporters are employed in the uptake of this ion in the *kdp*-defective *E*. *faecalis* JH2-2 strain and analyze the impact of the second messenger c-di-AMP on the activity of these proteins. Bioinformatics analyses were performed to show the distribution of four putative potassium transport systems identified in *E*. *faecalis* JH2-2: KtrAB, KtrAD, KimA, and Kup. In this study, we show that the *E*. *faecalis* Kup and KimA proteins are specific high affinity potassium transporters. We also demonstrate that Kup is inhibited by c-di-AMP *in vivo*. Moreover, genetic approaches were performed in the *E*. *faecalis* JH2-2 strain in order to characterize the multiple physiological roles of each potassium transporter system. We determined that KtrA is required to resist the growth at high pH and concentration of osmolites (NaCl, sorbitol). For the first time, we found evidence that Kup is also required to grow in complex medium at high pH under a limited K^+^ concentration and to resist osmotic stress.

## 2. Materials and methods

### 2.1. Bacterial strains and cultures

The bacterial strains used in this study are listed in Supplementary Table S1. *Escherichia coli* strain DH5α was used as an intermediate host for cloning, and *E*. *coli* EC101 was used as host for pBVGh constructs. *Escherichia coli* strains were grown at 37°C under aerobic conditions with vigorous shaking in Lysogeny Broth medium (LB). Potassium transporter deficient *E*. *coli* LB650 and LB2003 strains were cultivated in LB medium with the addition of 50 mM KCl or in minimal salts M9/M9mod medium supplemented with 50 mM KCl, unless otherwise stated. M9 medium contains 4 mM Na_2_HPO_4_, 22 mM KH_2_PO_4_, 18.5 mM NH_4_Cl, 1 mM MgSO_4_, 0.1 mM CaCl_2_, 0.5 μM FeCl_3_, 350 μM proline, 3 μM thiamine-di-chloride, 0.66% Casamino Acids, and 0.5% glucose or 0.2% glycerol as sources of carbon. Experiments with defined potassium concentrations were performed in M9mod, in which M9 potassium salts were replaced with equimolar quantities of sodium salts, and KCl was added as indicated. *Enterococcus faecalis* strains were routinely grown at 37°C without shaking in LB medium containing 0.5% w/v glucose (LBG). Alternatively, M17 medium (Oxoid) supplemented with 0.5% w/v glucose (M17G) and M17G supplemented with 0.5 M saccharose (SM17G) were employed when indicated. Experiments requiring low K^+^ medium were performed in mLBG, a modified LBG medium in which yeast extract was reduced from 0.5 to 0.025%; the K^+^ concentration of mLBG was less than 1 mM (Kawano et al., 2001). KCl, NaCl, and/or sorbitol were added to the growth media at the concentrations indicated in the figure legends. pH adjustments were made with addition of either HCl or NaOH. Suitable antibiotics were added to the media as selective agents when needed. For agar plates, 1.5% agar was added to the medium.

### 2.2. DNA manipulation

Plasmid DNA from *E*. *coli* cells was prepared with a Wizard Plus Minipreps DNA purification system (Promega). Chromosomal DNA of *E*. *faecalis* was extracted using Microbial DNA Kit (Macherey-Nagel). Treatment of DNA with restriction enzymes, T4 DNA ligase, and DNA polymerases was performed as recommended by the suppliers. DNA fragments were purified using the Wizard SV Gel and PCR Clean-Up System (Promega). DNA sequences were checked by sequencing in the World Meridian Venture Center, Macrogen’s sequencing service (Seoul, Korea). *Escherichia coli* cells were transformed using standard procedures (Sambrook et al., 1989). *Enterococcus faecalis* transformation was performed by electroporation according to the procedure of Dornan and Collins (1990).

### 2.3. Plasmid construction

Heterologous expression of the putative potassium transporter proteins in *E*. *coli* was approached as follows: the *kup* and *kimA* genes from *E*. *faecalis* JH2-2 were amplified using the oligonucleotide pairs GA1/GA2 and GA3/GA4, respectively. The PCR products were cloned between the BamHI and SalI sites of plasmid pWH844 to allow IPTG-inducible expression. The resulting plasmids were designated as pWH-*kup* and pWH-*kimA*. The pBAD33 and pWH844 expression vectors have compatible selection markers and origin of replications allowing the co-expression of potassium transporter genes (from pWH844) and the *cdaA* variants (from pBAD33; Gibhardt et al., 2019; Quintana et al., 2019).

### 2.4. Growth of *Escherichia coli* in minimal salt medium

Strains derived from *E*. *coli* LB650 (Δ*kdpABC5*, Δ*trkD1*, Δ*trkH*,Δ*ktrG*, Km^50^, and Cm^30^, see Supplementary Table S1) were propagated twice from −80°C stocks in M9mod medium supplemented with 1% glucose, the corresponding antibiotics, and 50 mM KCl. The overnight culture obtained was used to inoculate fresh M9mod medium supplemented with antibiotics, and 10 mM KCl for strains expressing the Kup and KimA proteins or 100 mM KCl for the strain expressing the empty vector control. At an OD_600_ of 0.5, cells were harvested. The pellet was re-suspended in the initial volume of fresh medium without KCl addition and incubated for 1 h at 37°C. Cultures were harvested again and washed three times with fresh medium. These samples were then used to inoculate fresh M9mod medium in microplates supplemented with 1% glucose, antibiotics, and different KCl concentrations. Microplates were incubated at 37°C with continuous orbital shaking and culture growth was monitored by measuring OD_600_ in 15-min intervals in a PowerWave™ XS Microplate reader (BioTek, BioTek Instrument Inc., Vermont, United States).

### 2.5. Co-expression of potassium transporters and diadenylate cyclases

Strain *E*. *coli* LB2003 (Δ*kdpABC5*, *kupD1*, and Δ*trkA*), a clean deficient in the gene encoding potassium transporter but sensible to antibiotic which allow the introduction of plasmids encoding kanamycin or chloramphenicol resistance determinants was co-transformed with pWH844 or derivatives and the pBAD33 derivatives (see Supplementary Table S1). This strain was inoculated from −80°C stocks in M9mod medium supplemented with 0.2% glycerol as the carbon source, corresponding antibiotics, and 10 mM KCl and propagated twice. At an OD_600_ of 0.5, cultures were harvested and re-suspended in same volume of M9mod medium containing 0.2% glycerol (no KCl added). Samples were incubated at 37°C for 1 h, after which two wash steps were performed. These washed samples were used for microplate inoculation supplemented with 0.075 mM KCl, and 0.05% L-arabinose when indicated. Antibiotics were added as appropriate. Microplates were incubated at 37°C with continuous orbital shaking and culture growth was monitored by measuring OD_600_ in 15-min intervals in a PowerWave™ XS Microplate reader (BioTek Instrument Inc., Vermont, United States).

### 2.6. Construction of *Enterococcus faecalis* potassium transporter defective strains and complementation of the mutants

Deletion of *kup*, *kimA*, and *ktrA* from *E*. *faecalis* JH2-2 was carried out using the thermosensitive vector pBVGh (Blancato and Magni, 2010). The oligonucleotides used for the amplification of upstream and downstream fragments of *kup*, *kimA*, and *ktrA* genes are indicated in Supplementary Table S1. Fragments were purified, digested, and ligated into the corresponding sites of the pBVGh vector. Cloned fragments were checked by sequencing. Finally, the protocol to generate the chromosomal deletion in *E*. *faecalis* was followed as previously described (Blancato and Magni, 2010). The Δ*kup* Δ*kimA*, Δ*kup* Δ*ktrA*, and Δ*kimA* Δ*ktrA* double mutants were obtained by eletroporating the pBVGh-*kimA* plasmid in the Δ*kup* and Δ*ktrA* single mutants, and the pBVGh-*ktrA* plasmid in the Δ*kup* single mutant. All gene deletions were confirmed by PCR sequencing using a pair of primers hybridizing in the adjacent up and downstream genes (Supplementary Table S1). The full-length *ktrA* gene was amplified by PCR using chromosomal DNA extracted from *E*. *facealis* JH2-2 as template and the pair of primers GA19/GA20 (Supplementary Table S1). The resulting fragment was purified, digested with the appropriate restriction enzymes, and ligated into the pBV153 vector giving rise to plasmid pBV-*ktrA*. The Δ*kup*Δ*ktrA* and Δ*kimA*Δ*ktrA* double mutants were complemented by the plasmid pBV-*ktrA*. Vector pBV153 was also transformed into the wild-type and the mutant strains as a control of any possible effect of plasmid transformation alone.

### 2.7. Growth of *Enterococcus faecalis* strains under stress conditions

In order to compare the growth curves of wild-type and the different potassium transporter mutant strains, *E*. *faecalis* was cultivated in microplates at 37°C. For this, overnight cultures grown in LBG medium were used to inoculate fresh LBG medium adjusted to different initial pH values, as indicated. The inoculums were diluted to an initial OD_600_ of 0.1. After the cell culture had reached the exponential growth phase, the cells were harvested and washed twice with fresh medium. These samples were then diluted to an initial OD_600_ of 0.1 in LBG or mLBG medium at different initial pH, and supplemented with 10 mM KCl when indicated. The OD_600_ was registered every 20 min in a PowerWave™ XS Microplate reader.

For osmotolerance assays, cells were cultured as described above except with the addition of either NaCl or sorbitol, and KCl when indicated, at the specified concentrations. The growth rates were calculated and plotted against the NaCl concentrations, which ranged between 0 and 8%.

Growth in urine or blood was analyzed as described previously (Martino et al., 2018). Infection and survival experiment*s* in *Galleria mellonella* were carried out as described (Terán et al., 2022).

### 2.8. Bioinformatic analysis

Genomic sequence data sets as well as predicted coding sequences of *E*. *faecalis* strains were retrieved from GenBank^1^ using Download Genomes tool.^2^ Datasets were composed of sequences submitted until 10 October 2020. Maximum likelihood phylogenetic trees were constructed as reported (Espariz et al., 2016). To counter-select potential paralogues, coverage and identity percentage cut-offs were set at 70% using GeM-Pro tool (Torres Manno et al., 2019). Metadata from NCBI biosample were obtained *via ad hoc* R script.^3^ Strains were classified based on these metadata in the categories such as clinical, commensal, environmental, or food sample.

For presence/absence analysis, the sequences of KimA, KupA, KtrA, KtrB, KtrD, KdpFABC, KdpDE, TrkHA, AtpIABCDFE, NtpFIKECGABDJ, and NapA proteins were used as query in TBLASTN (Blast +2.9.0) searches against *E*. *faecalis* genomes using as thresholds coverages > = 70% and identities > = 50%. In order to determine associations between protein-coding genes and metadata-assigned categories, enrichment statistical analyses were performed with PhyloLM V2.6 (Levy et al., 2018) using “logistic_IG10” method. The multiple comparisons for the statistical tests were corrected with Benjamini–Hochberg approach (Benjamini and Hochberg, 1995).

## 3. Results

### 3.1. KimA and Kup of *Enterococcus faecalis* encode high affinity potassium transporters

**Figure 2:**
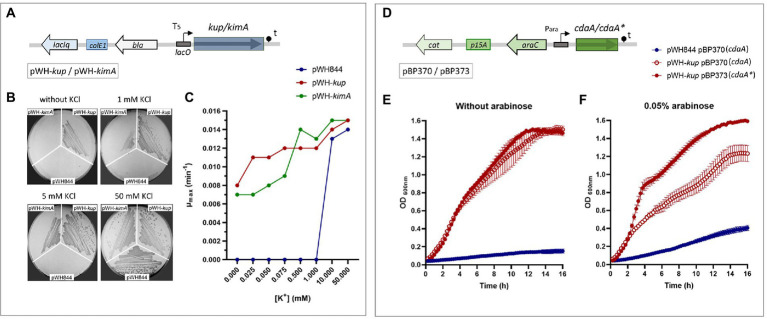
KimA and Kup of *Enterococcus faecalis* are potassium transporters. **(A)** pWH844 derived carrying full copy of Kup (pWH-*kup*) or KimA (pWH-*kimA*) used in this study. **(B)** *Escherichia coli* LB650 strains harboring plasmids pWH844, pWH-*kup*, and pWH-*kimA* were grown in M9-mod solid medium with or without supplementation of KCl (1, 5, or 50 mM). **(C)** Growth rate parameters determined at different KCl concentrations of LB650 derived strains, pWH844 (blue dot), pWH-*kup* (red dot), and pWH-*kimA* (green dot). Inhibition of Kup activity by c-di-AMP **(D)** pBAD derived carrying a copy of *cdaA^lmo^* (pBP370) or *cdaA*^lmo*^ (pBP373) used in this study. **(E,F)** *Escherichia coli* LB2003 harboring the plasmid combinations pWH844/pBP370 (blue dot), pWH-*kup*/pBP370 (empty red dot), and pWH-*kup*/pBP373 (full red dot), was cultivated in minimal salt M9mod medium, and supplemented without arabinose **(E)** or with arabinose 0.05% **(F)**.

Potassium transport systems present in *E*. *faecalis* JH2-2 (a natural deficient in the Kdp system strain) were identified based on homology with the proteins present in strain V583. Strain JH2-2 codes for the KtrAB and KtrAD channels and two members of the Kup family: Kup and KimA (Table 1). To test the functional role of the enterococcal proteins KimA and Kup, the *E*. *faecalis* JH2-2 *kup* and *kimA* genes were individually cloned in the pWH844 vector (Figure 2A). The resulting pWH-*kup* (*kup*) and pWH-*kimA* (*kimA*) plasmids were introduced in the *E*. *coli* strain LB650 that lacks the main potassium transport systems KdpABC, Kup (formerly TrkD, TrkH, and TrkG and is unable to grow at low K^+^ concentrations; Schlosser et al., 1995; Supplementary Table S1). The transformants were used to evaluate whether the expression of these enterococcal proteins could restore growth in minimal salt media when no K^+^ salts are added. As shown in Figure 2B, strain LB650 harboring the empty vector pWH844 was unable to grow in M9 minimal salt medium plates (M9mod), in which potassium salts were replaced by equimolar quantities of the respective sodium salts (see the section Materials and methods), without or in the presence of both 1 and 5 mM of KCl. Colonies of the triple mutant LB650 transformed with the vector pWH844 appear on the plates only when the concentration of KCl added reaches 50 mM (Figure 2B, colonies number 1 in each plate). *Escherichia coli* strain LB650/pWH-*kup* grows in M9mod independent of the addition of external K^+^ salt (Figure 2B, colonies number 2 in each plate), whereas strain LB650/pWH-*kimA* recovered ability to grow in M9mod in the presence of 1 mM KCl in solid medium (Figure 2B, colonies number 3 in each plate). In all cases, the cells were propagated in M9mod plates without IPTG induction. Thus, the basal expression of each of the enterococcal genes, due to readthrough of the *lac* promoter present in the vector pWH844, is already sufficient to allow potassium uptake and, consequently, growth of the complemented mutant strains.

**Table 1:** Potassium transport systems present in *Enterococcus faecalis* strains.

	KdpDE	KdpFABC	KtrA	KtrB	KtrD	KimA	Kup
V583	Ef0571-0570	Ef0566-0569	Ef2910	Ef0295	Ef2558	Ef0860	Ef0872
JH2-2	—	—	0994–2329	0994–2501	0994–2056	0994–0592	0994–0603

In addition, growth curves were determined in liquid M9mod medium with external additions of KCl to reach final concentrations between 0.025 and 50 mM. The growth parameters maximum optical density (OD_max_) and μ_max_ (Supplementary Table S2) were determined for the strains LB650/pWH844, LB650/pWH-*kup*, and LB650/pWH-*kimA*. Then, the μ_max_ were plotted against the potassium concentrations added to the medium (Figure 2C). The strains carrying the full copies of each enterococcal gene, *kup* or *kimA*, showed similar patterns in the variation of the values of μ_max_ vs. the K^+^ concentration. Both transporters allowed growth even at very low concentrations of KCl and, furthermore, it can be seen as μ_max_ increases as the concentration of K^+^ in the medium increases. However, the latter is not consistent with the OD_max_ values, as this parameter remains constant throughout the curve (Supplementary Table S2). The addition of the inducer IPTG in the medium produces a defect on the growth of the cells containing *kup* or *kimA* genes, that could be because overexpression of membrane proteins is often toxic and interferes with essential functions or because of an accumulation of toxic levels of potassium ions inside the cell (data not shown).

Recent studies have demonstrated that several members of the Kup transporter family (KimA from *B*. *subtilis* and *Listeria monocytogenes*, KupA and KupB from *L*. *lactis*) are negatively regulated by direct interaction with the nucleotide second messenger c-di-AMP (Gibhardt et al., 2019; Gundlach et al., 2019; Quintana et al., 2019). To test whether c-di-AMP impacts on the potassium transport activity of Kup and KimA, a co-expression system was established in the defective potassium transporter *E*. *coli* strain LB2003. Like *E*. *coli* LB650, this strain is deficient in the Trk, Kup, and Kdp potassium transport systems, hence is unable to grow in low K^+^ medium without complementation of a potassium transporter coding gene. A relevant fact is that *E*. *coli* lacks c-di-AMP synthesizing enzymes; therefore, the co-expression system to assess the phenotypic effect of c-di-AMP on the potassium transport systems KimA and Kup, can be carried out without interference of host-synthesized c-di-AMP. To control c-di-AMP recombinant production in *E*. *coli*, the diadenylate cyclase CdaA from *L*. *monocytogenes* was used. The CdaA*^Lmo^* (carrying the wild type gene of *cdaA*) and the inactive CdaA^Lmo*^variant D171N are encoded by the plasmids pBP370 and pBP373, respectively (Rosenberg et al., 2015). These plasmids are pBAD33 derivatives, allowing expression of genes under control of the arabinose P_ara_ promoter (Figure 2D), and compatible with pWH844 derivatives that were used to express the potassium transporters (Figure 2A). For the co-expression assays, the *E*. *coli* LB2003 derivative strains carrying pWH844 or derivatives as well as either of the two diadenylate cyclases encoding plasmids were grown in M9mod supplemented with KCl, and L-arabinose when indicated, at the specified concentrations (see the section Materials and methods).

Since in our previous growth experiments performed in *E*. *coli* LB2003 harboring a copy of *kimA* or *kup*, addition of 0.5 mM KCl to M9mod medium was sufficient for the strains to grow, this concentration was selected for the later co-expression assays. It is important to mention that at this potassium concentration, strain LB2003 harboring empty vector pWH844 was not able to grow (Figure 2E, blue circle). The growth patterns of the strains synthesizing Kup protein, carrying either the plasmid encoding the active CdaA*^Lmo^* (Figure 2E, empty red circle) or the inactive CdaA^Lmo*^ (Figure 2E, fill red circle) proteins were similar in the absence of the inducer (arabinose). By contrast, in the presence of 0.05% arabinose the growth of the strain synthesizing Kup was reduced when the functional diadenylate cyclase CdaA*^Lmo^* protein was co-produced, and thus in the presence of c-di-AMP (Figure 2F, empty red circle); however, no effect on the growth of the strain was observed when the inactive CdaA^Lmo*^ protein was co-produced, and thus in the absence of c-di-AMP (Figure 2F, fill red circle).

To test the possibility that high intracellular c-di-AMP concentrations or expression of the heterologous diadenylate cyclase affect growth of the strain co-producing Kup and the active CdaA*^Lmo^*, similar growth curves of *E*. *coli* LB2003 harboring the empty vector pWH844 and plasmid pBP370 or pBP373 were performed. In this assays, 50 mM KCl was added to the growth media, condition that allows growth of the bacteria. The growth of the strain carrying pWH844 as well as either of the two CdaA encoding plasmids was not reduced both in absence and presence of the inducer arabinose, and both strains exhibited similar growth phenotypes, as observed for the strain carrying the empty vector pWH844.

Similar experiments were performed to assess the effect of c-di-AMP on KimA. Despite different amounts of arabinose were added to de culture medium, the results obtained were unable to show evidence of the specific interaction between the second messenger and the transport system under study (data not shown).

### 3.2. Inactivation of genes coding for potassium transporter systems belonging to the Kup family in *Enterococcus faecalis* JH2-2

To investigate whether *kup* and *kimA* genes products are involved in K^+^ uptake in *E*. *faecalis*, simple Δ*kup* and Δ*kimA*, and double Δ*kup*Δ*kimA* mutants were generated using the chimeric vector pBVGh (Blancato and Magni, 2010). *Enterococcus faecalis* JH2-2 strain is able to grow in LBG or low K^+^ medium mLBG (Kawano et al., 2001) at broad pH values (initial pH 9.0, 7.0, and 5.0 units). In LBG medium, when the initial pH was fixed to 9.0, enterococcal cells reached the maximal OD_600_ and growth rate (1.22 OD and 0.81 h^−1^, respectively; Supplementary Figure S1). At this pH, in mLBG a decrease of the growth parameters was observed (0.67 OD and 0.46 h^−1^, respectively; Supplementary Figure S1). At neutral initial pH (7 unit), similar patterns of growth were observed in both media, LB (0.63 OD and 0.88 h^−1^, respectively) and mLB (0.54 OD and 0.61 h^−1^, respectively). The reduction of the biomass generation could be due to the rapid acidification of the medium in a batch culture (Supplementary Figure S1). At acidic condition (pH 5.0), a significative reduction in the growth parameters was observed in LB and mLB media (OD = 0.53, growth rate 0.49 h^−1^ and OD = 0.36, growth rate 0.19 h^−1^, respectively; Supplementary Figure S1). The growth parameters of each enterococcal mutant strain (Δ*kup* and Δ*kimA*, and Δ*kup* Δ*kimA*) were analyzed by testing growth in both LBG or mLBG, and at the same pH values (9.0, 7.0, and 5.0). No differences were observed in the values of biomass and growth rate of the single and double mutants compared to the wild-type strain *E*. *faecalis* JH2-2 (Supplementary Figure S2).

### 3.3. Effect of Kup and KimA gene deletion on deficient strain in the Ktr channel systems

**Figure 3:**
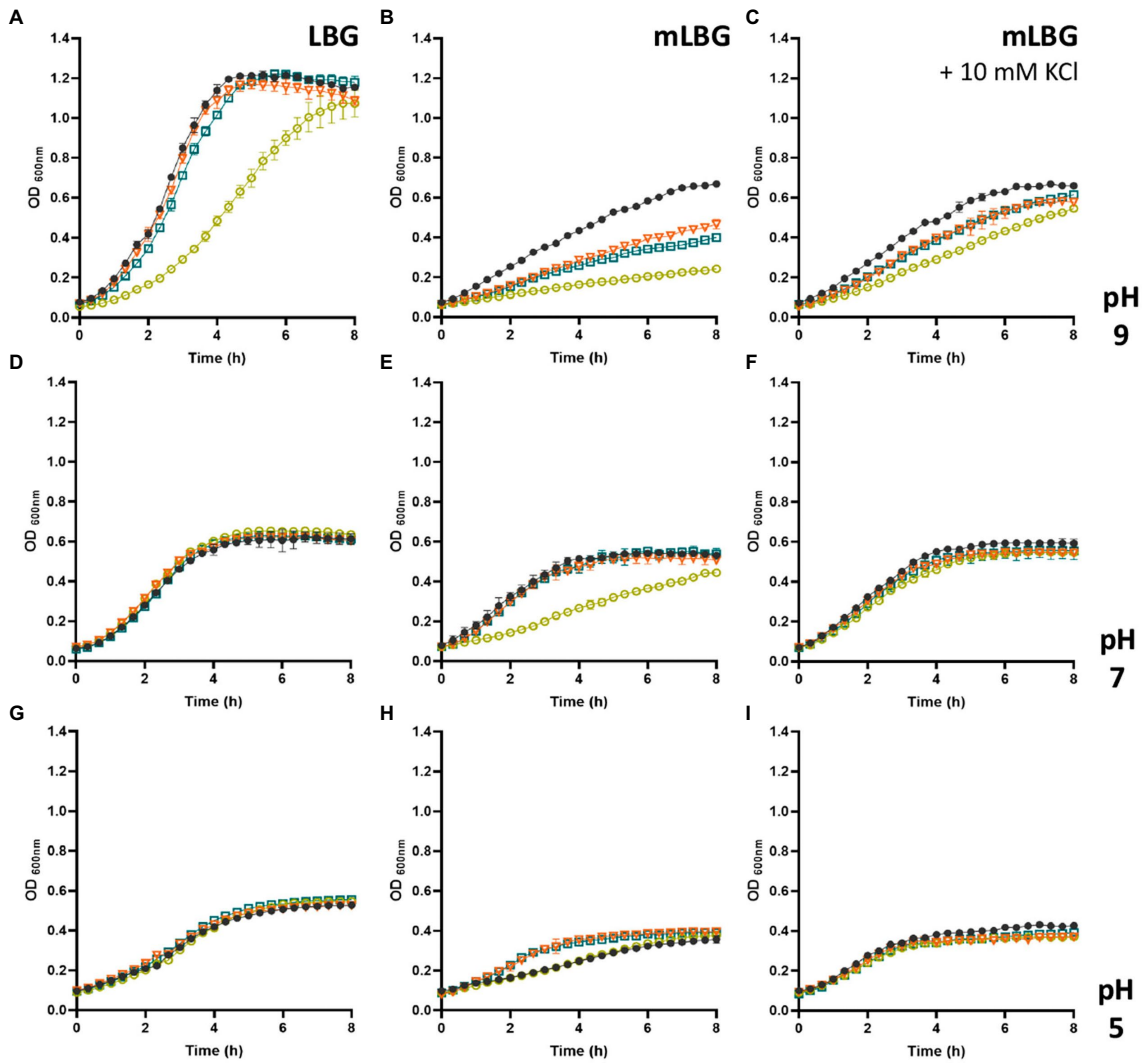
Growth curves of *Enterococcus faecalis* JH2-2 mutants under different conditions. Wild type (black dot), Δ*ktrA* (light blue square), Δ*kup*Δ*ktrA* (yellow circle), and Δ*ktimA*Δ*ktrA* (orange triangle) strains were grown in LBG **(A,D,G)**, mLBG **(B,E,H)**, or mLGB medium supplemented with 10 mM KCl **(C,F,I)**. Initial pH was fixed at 9.0 units **(A–C)**, 7.0 **(D–F)**, or 5.0 **(G–I)**.

Taking into account the results presented above and the pivotal role of the Ktr channel systems in *E*. *faecalis*, different Ktr deficient strains were generated. Since previous studies in other related microorganisms have demonstrated that one ion-transporting protein (KtrB or KtrD) and the KtrA regulator function together in K^+^ uptake (Holtmann et al., 2003; Gries et al., 2013), the suspected major function of Ktr channel was tested by generating a deletion of the *ktrA* gene. A series of different K^+^-transporter systems deficient strains was constructed (Δ*ktrA*, Δ*ktrA* Δ*kup*, and Δ*ktrA* Δ*kimA*). The rates of growth of the wild type and the *ktrA*-derived mutants were compared in LB, and mLB at different pH values (Figure 3). In LB medium, only the double mutant Δ*kup* Δ*ktrA* strain shows reduced growth at alkaline pH (Figure 3A, yellow circle), whereas no other mutant showed phenotype at the other pHs in this medium (Figure 3D, pH 7.0 and 2G, pH 5.0). When the concentration of K^+^ in the complex medium was reduced (mLB), delayed growth was observed at alkaline conditions (pH 9.0) for all *ktrA* mutants, which was more severe for the double mutant Δ*kup* Δ*ktrA* (Figure 3B, yellow circle). In the same medium at neutral pH, this growth defect was observed only for the Δ*kup* Δ*ktrA* double mutant (Figure 3E, yellow circle). At acidic pH 5.0 in mLB, no growth alterations were observed for the mutant strains as compared to the JH2-2 parent strain (Figure 3H). Growth deficiencies of the KtrA mutants were restored to wild type phenotype by the addition of potassium (10 mM) to the growth medium (Figures 3C,F), strongly suggesting the inability of the mutant strains to concentrate K^+^ ions inside the cell.

### 3.4. Comparison of osmotic response of the *Enterococcus faecalis* potassium transporter deficient strains

**Figure 4:**
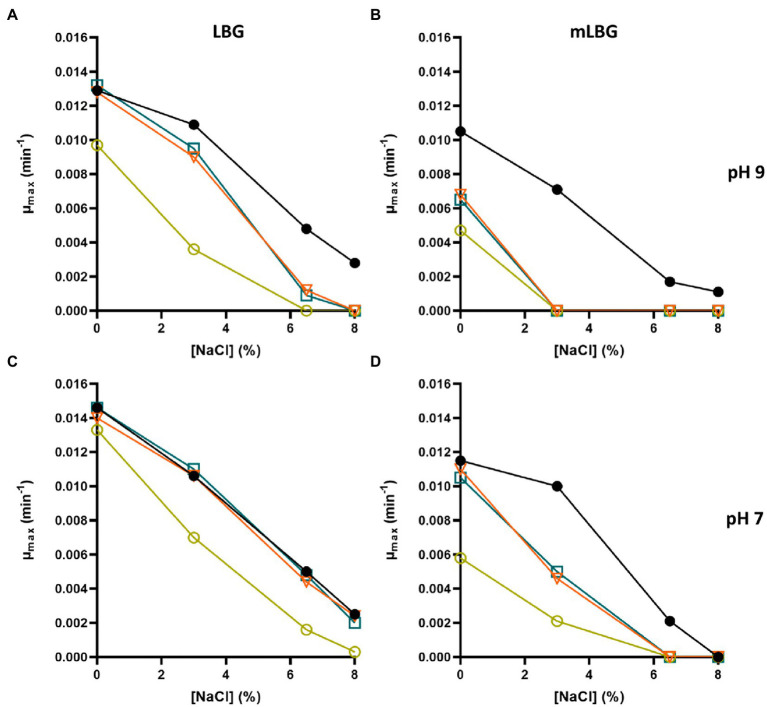
Inhibition of growth rate of *Enterococcus faecalis* JH2-2 mutants by NaCl. Wild type (black dot), Δ*ktrA* (light blue square), Δ*kup*Δ*ktrA* (yellow circle), and Δ*ktimA*Δ*ktrA* (orange triangle) strains were grown in LBG **(A,C)** and mLBG medium **(B,D)** at initial pH 9.0 (top) or 7.0 (low) in the presence of increasing concentrations of NaCl.

**Figure 5:**
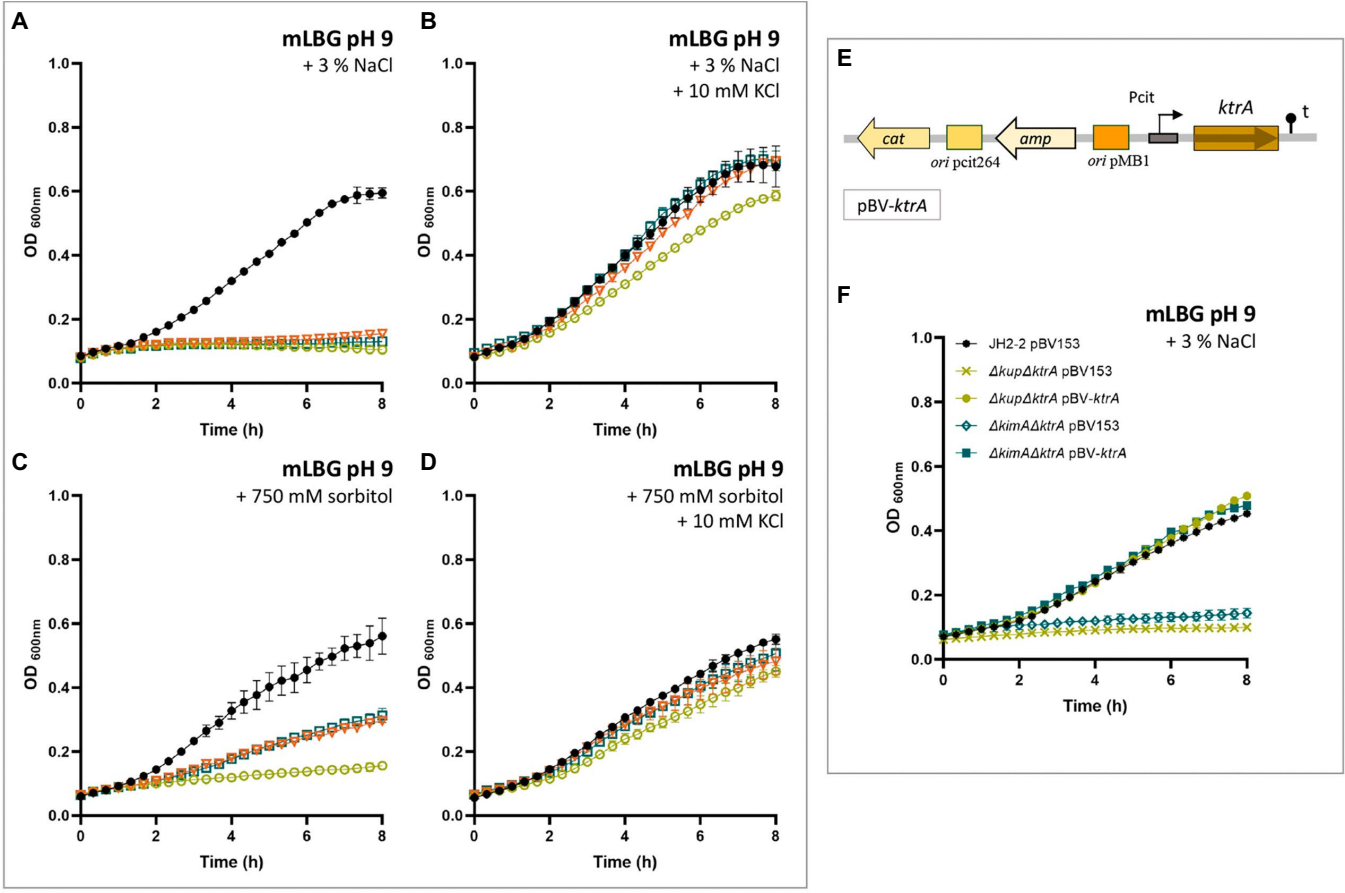
KtrA contributes to the osmotic response in *Enterococcus faecalis*. Wild type (black dot), Δ*ktrA* (light blue square), Δ*kup*Δ*ktrA* (yellow circle), and Δ*ktimA*Δ*ktrA* (orange triangle) strains were grown in mLBG at alkaline initial pH and supplemented with 3% NaCl **(A)** or 750 mM sorbitol **(C)**. In both growth conditions, the addition of 10 mM KCl (**B** and **D**, respectively) completely restored defective strains growth to near wild-type levels. **(E)** Schematic representation of the plasmid pBV-*ktrA* used for complementation of a full copy of the wild type *ktrA* gene. **(F)** Expression of *ktrA* from a pBV153 fully complemented the growth defects of the KtrA-mutant strains in mLBG at alkaline initial pH and supplemented with 3% NaCl, and in all conditions tested in this study (data not shown).

As mentioned above, *E*. *faecalis* is able to resist high concentrations of NaCl. The first response to cope with hyperosmotic stress is K^+^ accumulation *via* massive uptake (Stülke and Krüger, 2020). The effect of salt compound on the growth of the parental and derived potassium transporters deficient strains of *E*. *faecalis* JH2-2 was analyzed in LBG and mLBG at different starting pH (9.0, 7.0, and 5.0) in the presence of up to 8% NaCl. As shown in Figure 4, during growth under alkaline stress conditions (pH 9.0), the toxic effect of NaCl addition is observed in both media, LB and LBm, for all *ktrA* mutants (Δ*ktrA*, Δ*kup* Δ*ktrA*, and Δ*kimA* Δ*ktrA*). Particularly, in LBG, the double mutant strain Δ*kup* Δ*ktrA* exhibited a more pronounced defect in growth as compared to wild type JH2-2 strain (Figure 4A, yellow circle). When the content of K^+^ in the medium is reduced but the pH remained the same, all defective strains were unable to grow at the lowest amount of the salt added (3% NaCl; Figures 4B, 5A), while the JH2-2 strain resisted up to 6% NaCl. As expected, these defects in the growth of all *ktrA* mutant strains were restored by addition of 10 mM KCl (Figure 5B). Growth in LB medium at neutral conditions (pH 7.0) with the addition of increasing concentrations of NaCl (from 0 to 8%), had similar effects for the wild type and the Δ*ktrA* and Δ*kimA* Δ*ktrA* mutant strains, however, a clear inhibition was observed for the double mutant Δ*kup* Δ*ktrA* (Figure 4C). When strains were grown in mLBG and neutral pH, again all *ktrA* mutants exhibited defective growth or sensitivity to the osmotic stressor NaCl, and normal growth was observed with the addition of 10 mM KCl (data not shown). Finally, at low pH condition (5.0), the treatment with NaCl led to drastically reduced growth parameters for all the tested strains; thus, the osmotic effect was not evident (data not shown).

Additionally, the ability of the mutant strains to grow in low K^+^ medium mLB supplemented with a low molecular weight osmolyte, different than NaCl, was tested to guarantee that the NaCl-induced effects on growth reported here were due to an osmotic effect and not to an ionic one. Thus, the growth of *ktrA* deficient mutant strains of *E*. *facecalis* was examined at high concentrations of sorbitol. Both in neutral and alkaline conditions, the addition of 750 mM sorbitol to mLB medium had a severe impact on growth of the Δ*ktrA*, Δ*kimA* Δ*ktrA*, and Δ*kup* Δ*ktrA* strains as compared to the wild type strain (Figure 5C). Similar to the growth in presence of 3% NaCl, addition of 10 mM KCl to the culture medium restored growth of the mutant strains (Figure 5D).

Finally, defective phenotypes found in the Δ*ktrA*-containing mutants were analyzed by complementation with a full copy of the wild type *ktrA* gene, by use of the plasmid vector pBV153 (Figure 5E). Since the phenotype of the single mutant in KtrA and the double mutant in KtrA and KimA was the same in all the conditions examined, the complementation was performed only with the *ktrA* gene on the double mutant strains. Expression of *ktrA* rescued the growth defect of Δ*kimA* Δ*ktrA* and Δ*kup* Δ*ktrA* mutants to wild-type levels in mLBG under alkaline constitions (pH 9.0) and in the presence of 3% NaCl (Figure 5F). This observation demonstrates that the KtrA protein is indeed required to allow growth of *E*. *faecalis* under alkaline conditions or in the presence of NaCl.

### 3.5. Diversity of K^+^ transporters in *Enterococcus* species

**Figure 6:**
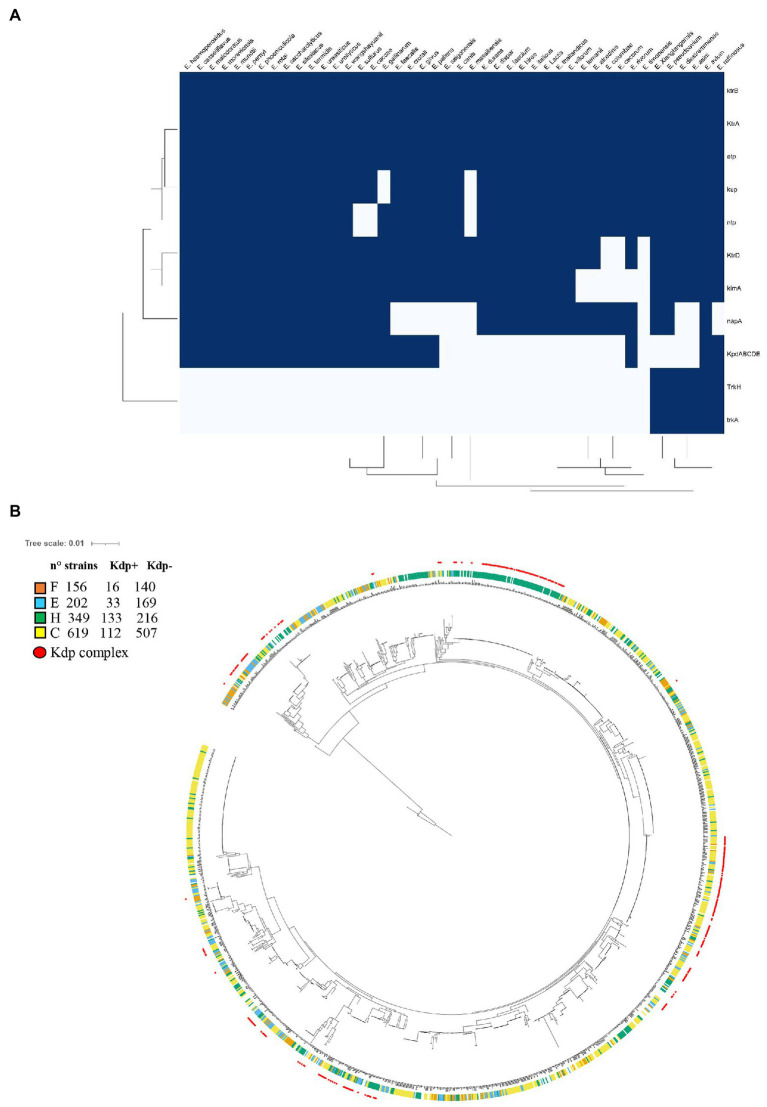
**(A)** Heatmap showing the presence of K^+^ transporters and related ATPases in *Enterococcus* species. A presence-absence matrix was constructed and Distance-based clustering was applied. Dark blue indicates presence of the genes analyzed. **(B)** Analysis of the presence of *kdp* genes and the phylogenomic relationships among *E*. *faecalis* members. 34 common ancestral genes were individually aligned, concatenated, and trimmed resulting in a final alignment containing a total of 4,726 residues. The evolutionary history of the 1,510 *E*. *faecalis* members was inferred with RAxML algorithm (Stamatakis, 2014) and displayed and annotated using iTOL (Letunic and Bork, 2011). The inner circles colors indicate the source of isolation; F, food (orange); C, commensal (yellow); E, environmental (light blue); and H, clinical (green). The red outside circles indicate the presence of the *kdp* genes within the strains.

Orthologs of the Kdp pump, Ktr/Trk channels, and Kup symporter family (Stülke and Krüger, 2020; Tascón et al., 2020) were collected from the species with finished whole sequence genomes (NCBI) of the family that include the genus *Enterococcus* (44 species). As shown in the Figure 6A, distribution of the *kdp* operon is limited to 24 of the 44 species of *Enterococcus* analyzed. *kup* was found in 42/44 species studied of *Enterococcus* (mutated in *Enterococcus massiliensis*), whereas *kimA* is present in 38/44 species of *Enterococcus*. In the case of *ktr* genes, both genes encoding for the cytosolic component KtrA and for the membrane component of the system, KtrB, were found encoded in all 44 species analyzed. The *ktrD* gene encoding for the membrane component was found in 41 species. On the other hand, the *trkAH* operon, encoding the cytoplasmatic component TrkA (455 residues) and the membrane component TrkH (479 residues), was found disseminated in only a few species (*Enterococcus asini*, *Enterococcus avium*, *Enterococcus diestrammaneae*, *Enterococcus raffinosus*, *Enterococcus pseudoavium*, and *Enterococcus xiangfangensis*).

In addition to the K^+^ transporters, other transporters associated to ion fluxes were sought in *Enterococcus*. Such is the case of the F_0_F_1_-ATPase complex, a well conserved proton translocator which has an essential function in the establishment of the proton motive force (PFM) and, therefore, it is related to the mechanisms of resistance to low pH. Also, the presence of Na^+^ transporters, which are involved in the alkaline and osmotic resistance, was identified. The results showed that the *ntp* operon, which encodes for the F_0_F_1_-ATPase complex, is widespread in *Enterococcus* species (41/44), with few exceptions (*Enterococcus canis*, *E*. *massilensis*, *and Enterococcus sulfureus*), while *napA*, encoding the Na^+^ symporter NapA, is present in 33 species of the 44 analyzed.

Finally, considering that the expression of the Kdp system has been studied in *E*. *faecalis* V583, and that the induction of this potassium transporter under alkaline and NaCl stress conditions was reported (Solheim et al., 2014), we hypothesized that the requirement of the Kdp system could be involved in the pathogenesis of clinical strain. Though KimA, Kup, and Ktr transporters are homogeneously distributed in nearly all *E*. *faecalis* strains, the KdpFABC complex and the regulatory KdpDE system were found in only 319 strains out of a total of 1,510 complete genome sequences analyzed (21%) of these species (Figure 6B). The latter indicates that, as in other species, these genes do not belong to the gene core in *E*. *faecalis*. Then, we classified the strains and tested the hypothesis that the presence of *kdp* genes is correlated with pathogenicity. The phylogenetic tree depicted in Figure 6B showed the existence of clusters of strains with high phylogenetic signal (very related phylogenetically) that have similar *kdp* occurrence. Nevertheless, using phylogeny-aware methods, we observed that the presence of *kdp* genes was significantly positively correlated with *E*. *faecalis* of clinical origin (*p* value of 2.76 E^−11^). Taking into account the relevance of the robust physiology of *E*. *faecalis*, which allows it to persist and thrive in harsh environments that include acid, oxidative and hyperosmotic stress, for its pathogenic potential (Solheim et al., 2014), we investigated whether K^+^ transporter systems contribute to the pathogenesis of the natural Kdp defective strain JH2-2. Notably, deletion of the potassium transport systems Kup, KimA, or Ktr in *E*. *faecalis* JH2-2 did not affect growth and survival in animal fluids associated to common diseases caused by this bacterium (blood and urine) nor the infection and virulence in *Galleria Mellonella* model (Supplementary Figures S3, S4).

## 4. Discussion

In this study two members of the KUP family, Kup and KimA, present in *E*. *faecalis* were identified and characterized. Our work demonstrates that both transporters possess a high affinity for the K^+^ ion, similar to family members described in *L*. *lactis* or *B*. *subtilis* (Gundlach et al., 2019; Quintana et al., 2019). The inhibition of K^+^ transport by the second messenger c-di-AMP was demonstrated, by *in vivo* studies in a c-di-AMP producing *E*. *coli* strain (Figure 2F), for the Kup protein, however, it could not be demonstrated for KimA. In Firmicutes, as mentioned above, regulation of potassium transport by the second messenger c-di-AMP was demonstrated for several members of this phylum; this is the case for the control of the activity of Kup from *L*. *lactis*, KimA from *B*. *subtilis* and *L*. *monocytogenes*, and KtrA/C from *B*. *subtilis*, *L*. *monocytogenes*, *S*. *aureus*, *S*. *pneumoniae*, and *S*. *mutans*. Considering the high similarity among orthologous transporters in this group of related bacteria, it is strongly suggested that K^+^ homeostasis in *E*. *faecalis* could be controlled by the intracellular c-di-AMP concentration (Figure 1). Also, as stated above, disruptions in both the potassium uptake and the c-di-AMP homeostasis can affect the virulence potential of bacterial pathogens. Recent studies with *E*. *faecalis* strain OG1RF (Kdp^+^ strain) revealed that variations in the intracellular levels of c-di-AMP modify its pathogenic potential (Kundra et al., 2021). In our study, no differences concerning the growth either in animal fluids or in the *G*. *mellonella* insect model were detected by using simple and double mutants in the different K^+^ transporters (Kup, KimA, and Ktr systems) present in *E*. *faecalis* JH2-2. This observation differs to findings in other opportunistic pathogen such *S*. *aureus*, where a KtrA deficient strain showed a deficiency in the capacity to infect mice (Gries et al., 2013). Here, it is important to mention that, in this study we were unable to obtain a K^+^ transporter-free triple mutant (Δ*kup* Δ*kimA* Δ*ktrA*) using our gene deletion methodology. Therefore, although further studies will be required, this impossibility suggests that at least one potassium transport system is necessary and sufficient to satisfy the demand for this ion in the cellular interior, in the media and conditions studied. On the other hand, a detailed analysis of the distribution of transport systems in clinical and non-clinical isolates of *E*. *faecalis* showed that the *kdp* genes were enriched significantly in clinical isolate samples.

Given that the robust nature of *E*. *faecalis* facilitates its tolerance to hostile environments, and the fact that bacterial cells are known to import K^+^ to help resist and survive under various stressful conditions, growth of simple and double potassium transport mutants derived from *E*. *faecalis* JH2-2 parental strain was assayed under different conditions. Interestingly, the inactivation of KtrA - dependent K^+^ channels contribute to the sensitivity to both alkaline growth condition and osmotic stress. These phenotypes were increased in a low K^+^ medium, and recovered by the external addition of potassium salt or by the introduction of a plasmid carrying a full copy of the wild type *ktrA* gene. We also found that inactivation of the K^+^ transporter Kup, but not KimA, in the KtrA—deficient mutant strain increases both the sensitivity to alkaline stress and the toxicity by NaCl. From the latter data, we propose that the Ktr system plays an important role in the ability of *E*. *faecalis* to adapt to hyperosmotic stress, and that Kup functions secondarily to Ktr. However, altogether the growth assays of the potassium transporter defective strains indicate that *E*. *faecalis* uses different transporters systems with capacity to replace one to the others in different stress conditions, such as low K^+^, extreme pH, and salt concentrations.

Based on the results of this study, we propose a general mechanism of *Enterococcus* resistance, which as shown in Figure 1 involves the expression of different ATPases for the movement of the essential ions. Proton extrusion is carried out by the F_0_F_1_ ATPase complex, which is functional at neutral and acidic pH. Sodium ions are pumped outside cells by the V_0_V_1_ ATPase complex, which is active at low H^+^ concentrations. Concerning to the homeostasis of K^+^, multiple channels and transporters are present in *E*. *faecalis*, but the specific physiological roles of each transporter are poorly studied. Diversity analysis of the potassium transporters performed in the *Enterococcus* species revealed that the channels KtrA/B is the most widely disseminated of the K^+^ transporters. The two members of the H^+^/K^+^ symporter KUP family, Kup and KimA, described here, are widely disseminated in the *Enterococcus* genera. Taking into account how the ion flux of H^+^, Na^+^, and K^+^ involved in alkaline stress occurs in this microorganism (Figure 1), and the results obtained in this work with respect to the mutant strains in potassium transport, we hypothesize that the KtrAB system is involved in the symport of K^+^ and Na^+^, and function together, under alkaline pH growth, with the Na^+^ ATPase, responsible to expel out the Na^+^. In addition, we found that Kup could mediate the intracellular import of K^+^ and H^+^, which could contribute to not only the homeostasis of potassium, but also to hold protons inside the cells. This participation of the Kup transporter in alkaline conditions is highly relevant, and its contribution could be that this protein can transport K^+^ ions with H^+^, thus contributing not only to K^+^ uptake but also to the acidification of the cytoplasm in extreme alkaline conditions.

## 5. Conclusion

*Enterococcus faecalis* is able to resist and grow in different conditions. By using genetic techniques, we conclude that KtrA is required for the functionality of the uptake system in alkaline and high osmolarity, in this condition also Kup increase the resistance to this stress conditions to the concentration of K^+^. Finally, simple or double mutans in the K^+^ transporter did not produce significative modification in the virulence of the strains.

## Data availability statement

The original contributions presented in the study are included in the article/Supplementary material, further inquiries can be directed to the corresponding author.

## Author contributions

GA done experimental design and performed experiments. FG performed experiments. MT contributed to bioinformatic analysis. ME contributed to experimental design and bioinformatic analysis. VB designed and performed experiments and bioinformatic analysis. JS designed experiments. CM designed experiments and performed bioinformatic analysis. All authors contributed to the article and approved the submitted version.

## Funding

This work was supported by Ministerio de Ciencia y Tecnologia PICT 2020–3227 (Prest BID) and PIP 11220200101356CO, CONICET.

## Conflict of interest

The authors declare that the research was conducted in the absence of any commercial or financial relationships that could be construed as a potential conflict of interest.

## Publisher’s note

All claims expressed in this article are solely those of the authors and do not necessarily represent those of their affiliated organizations, or those of the publisher, the editors and the reviewers. Any product that may be evaluated in this article, or claim that may be made by its manufacturer, is not guaranteed or endorsed by the publisher.

## Supplementary material

The Supplementary material for this article can be found online at: https://www.frontiersin.org/articles/10.3389/fmicb.2023.1117684/full#supplementary-material

## Footnotes

^1^ ftp.ncbi.nlm.nih.gov/genomes/

^2^ https://github.com/torresmanno/Download_Genomes

^3^ https://github.com/torresmanno/Acciarri_etal_Rscript
