# Supplementary material for: Redundant potassium transporter systems guarantee the survival of Enterococcus faecalis under stress conditions
Source: Front Microbiol. 2023 Feb 8;14:1117684. doi: 10.3389/fmicb.2023.1117684 (PMC9945522; doi:10.3389/fmicb.2023.1117684)
Supplement: Supplementary file 1 [file Table_1.DOCX]

**Table S1.** Bacterial, plasmids and oligonucleotides used in this study

| **Bacterial Strain** | **Genotype** | **Reference** |
| --- | --- | --- |
| ***E. coli* strains** |  |  |
| LB650 | *∆kdpABC5* *∆trkH ∆trkG* ; Km^R^; Cm^R^ | (Schlosser et al., 1995) |
| LB2003 | *∆kdpABC5 kupD1 ∆trkA* | (Stumpe and Bakker, 1997)(Stumpe and Bakker, 1997) |
| LB08 | LB650 - pWH844; Km^R^, Cm^R^, Amp^R^ | (Quintana et al., 2019) |
| LB650/pWH-*kup* | LB650 - pWH-*kup*; Km^R^, Cm^R^, Amp^R^ | This work |
| LB650/pWH-*kimA* | LB650 - pWH-*kimA*; Km^R^, Cm^R^, Amp^R^ | This work |
| 0380 | LB2003 - pWH844 and pBP370; Cm^R^, Amp^R^ | (Quintana et al., 2019) |
| 0383 | LB2003 - pWH844 and pBP373; Cm^R^, Amp^R^ | (Quintana et al., 2019) |
| 0380-*kup* | LB2003 - pWH-*kup* and pBP370; Cm^R^, Amp^R^ | This work |
| 0383-*kup* | LB2003 - pWH-*kup* and pBP373; Cm^R^, Amp^R^ | This work |
| 0380-*kimA* | LB2003 - pWH-*kimA* and pBP370; Cm^R^, Amp^R^ | This work |
| 0383-*kimA* | LB2003 - pWH-*kimA* and pBP373; Cm^R^, Amp^R^ | This work |
| EC101 | *E. coli* JM101 derived. *repA*^+^ (from pWV01 integrated in chromosome); Km^R^ | (Law et al., 1995) |
| DH5α | Δ*(lacZ)M15 gyrA96 recA1 relA1 endA1 thi-1 hsdR17* | (Hanahan, 1983) |

| ***E. faecalis* strains** |  |  |
| --- | --- | --- |
| JH2-2 | Cit^+^; Rif^R^; Fus^R^; plasmid free | (Jacob and Hobbs, 1974) |
| Δ*kup* | JH2-2 Δ*kup* | This work |
| Δ*kimA* | JH2-2 Δ*kimA* | This work |
| Δ*ktrA* | JH2-2 Δ*ktrA* | This work |
| Δ*kup*Δ*kimA* | JH2-2 Δ*kup*Δ*kimA* | This work |
| Δ*kup*Δ*ktrA* | JH2-2 Δ*kup*Δ*ktrA* | This work |
| Δ*kimA*Δ*ktrA* | JH2-2 Δ*kimA*Δ*ktrA* | This work |
| JH2-2 pBV153 | JH2-2 strain carrying pBV153 vector | This work |
| Δ*kup*Δ*ktrA* pBV153 | Δ*kup*Δ*ktrA* strain carrying pBV153 vector | This work |
| Δ*kimA*Δ*ktrA* pBV153 | Δ*kimA*Δ*ktrA* strain carrying pBV153 vector | This work |
| Δ*kup*Δ*ktrA* pBV-*ktrA* | Δ*kup*Δ*ktrA* strain carrying pBV-*ktrA plasmid* | This work |
| Δ*kimA*Δ*ktrA* pBV-*ktrA* | Δ*kimA*Δ*ktrA* strain carrying pBV-*ktrA plasmid* | This work |

| **Plasmid** | **Description** | **Reference** |
| --- | --- | --- |
| pWH844 | ColE1 derived carrying *lacI* and IPTG inducible promoter; Amp^R^ | (Schirmer et al., 1997) |
| pWH-*kup* | pWH844-*kup*; Amp^R^ | This work |
| pWH-*kimA* | pWH844- *kimA*; Amp^R^ | This work |
| pBP370 | pBAD33-*cdaA*^lmo^; Cm^R^ | (Quintana et al., 2019) |
| pBP373 | pBAD33-*cda*A^lmo^ D171N; Cm^R^ | (Quintana et al., 2019) |
| pBVGh | Thermosensitive vector for gene deletion Em^R^ | (Blancato and Magni, 2010) |
| pBVGh-*kup* | pBVGh derivade carrying up-down *kup* fragment | This work |
| pBVGh-*kimA* | pBVGh derivade carrying up-down *kimA* fragment | This work |
| pBVGh-*ktrA* | pBVGh derivade carrying up-down *ktrA* fragment | This work |
| pBV153 | Expression vector derived from pBM01 containing chromosomal pH-controllable promoter region Pcit and NdeI cloning site, Cm^R^ | (Marelli and Magni, 2010) |
| pBV-*ktrA* | pBV153 derivade carrying *ktrA* | This work |

Km, Kanamycin 50 μg/ml; Cm, Chloranphenicol 30 μg/ml; Amp, Ampicillin 100 μg/ml.

| **Oligonucleotide** | **Sequence** | **Purpose** |
| --- | --- | --- |
| GA1 | AAAGGATCCGTGTTACACAAAGCAGAGGGG | (Fwd) *kup* - *E. faecalis* JH2-2 |
| GA2 | TTTGTCGACATTTCTTCTATTTATGAACGATTCTTTC | (Rv) *kup* - *E. faecalis* JH2-2 |
| GA3 | AAAGGATCCATGGCGGATTTAAAACGACTG | (Fwd) *kimA* - *E. faecalis* JH2-2 |
| GA4 | TTTGTCGACATAGCAGACTTTTTTTTATTTTTTCAAGTG | (Rv) *kimA* - *E. faecalis* JH2-2 |
| GA5 | ATGCCATGGCTATGAAGTTGGACAAAC | (Fw) Up.*kup* - *E. faecalis* JH2-2 |
| GA6 | CCTATCACCTCAAATGGTTCGCTGCATTAGTCAAAAAGCGC | (Rv) Up.*kup* - *E. faecalis* JH2-2 |
| GA7 | CAGCGAACCATTTGAGGTGATAGGGTCCTTGAGATACATGGC | (Fw) Down.*kup* - *E. faecalis* JH2-2 |
| GA8 | ACCCCATGGTACTCAGTTGATGGTACG | (Rv) Down.*kup* - *E. faecalis* JH2-2 |
| GA9 | ACACAACAGTAGATTGGC | (Rv) Check.*kup* - *E. faecalis* JH2-2 |
| GA10 | CAGTGAATTTGTATGACC | (Fw) Up.*kimA* - *E. faecalis* JH2-2 |
| GA11 | CCTATCACCTCAAATGGTTCGCTGTTTCCCAATGAACAGTC | (Rv) Up.*kimA* - *E. faecalis* JH2-2 |
| GA12 | CAGCGAACCATTTGAGGTGATAGGTGCTATTTTTTGATGGC | (Fw) Down*.kimA* - *E. faecalis* JH2-2 |
| GA13 | CCACCATGGACACTTAATTCCGTCAGC | (Rv) Down.*kimA* - *E. faecalis* JH2-2 |
| GA14 | CAGCGAACCATTTGAGGTGATAGGATTTCTTCTGACCGC | (Fw) Up.*ktrA* - *E. faecalis* JH2-2 |
| GA15 | ATGCCATGGTGATGGATTTGGTAATGG | (Rv) Up.*ktrA* - *E. faecalis* JH2-2 |
| GA16 | AGCCCATGGAATAGTCCTACGCAC | (Fw) Down.*ktrA* - *E. faecalis* JH2-2 |
| GA17 | CCTATCACCTCAAATGGTTCGCTGTGAATGGCTAAGCAC | (Rv) Down.*ktrA* - *E. faecalis* JH2-2 |
| GA18 | TATTTCCTCCACACTTCAG | (Fw) Check.*ktrA* - *E. faecalis* JH2-2 |
| GA19 | ATAAAAGTTATTAGGAAAGAAGACATATGAAACAAAATTTTGCAATTATTG | (Fwd) *ktrA* - *E. faecalis* JH2-2 |
| GA20 | GAATTCACTAGTGATTGATCAACCATATGTTAGCCATTCATTTTTTC | (Rv) *ktrA*- *E. faecalis* JH2-2 |

**Table S2.** Growth parameters OD_max_ and μ_max_ for *E. coli* LB650 derivative strains.

|  | LB650/pWH844 | | LB650/pWH-*kup* | | LB650/pWH-*kimA* | |
| --- | --- | --- | --- | --- | --- | --- |
| [K^+^] (mM) | OD_max_ | μ_max_ (min ^-1^) | OD_max_ | μ_max_ (min ^-1^) | OD_max_ | μ_max_ (min ^-1^) |
| 0 | 0,11 | 0,000 | 0,80 | 0,008 | 1,02 | 0,007 |
| 0,025 | 0,07 | 0,000 | 0,80 | 0,011 | 0,98 | 0,007 |
| 0,05 | 0,09 | 0,000 | 0,80 | 0,011 | 0,98 | 0,008 |
| 0,075 | 0,10 | 0,000 | 0,80 | 0,012 | 0,98 | 0,009 |
| 0,5 | 0,12 | 0,000 | 0,80 | 0,012 | 0,98 | 0,014 |
| 1 | 0,16 | 0,000 | 0,80 | 0,012 | 0,98 | 0,013 |
| 10 | 1,48 | 0,013 | 0,80 | 0,014 | 0,86 | 0,015 |
| 50 | 2,10 | 0,014 | 1,00 | 0,015 | 0,82 | 0,015 |

Blancato, V. S., and Magni, C. (2010). A chimeric vector for efficient chromosomal modification in enterococcus faecalis and other lactic acid bacteria. *Lett. Appl. Microbiol.* doi: 10.1111/j.1472-765X.2010.02815.x.

Hanahan, D. (1983). Studies on transformation of Escherichia coli with plasmids. *J. Mol. Biol.* 166. doi: 10.1016/S0022-2836(83)80284-8.

Jacob, A. E., and Hobbs, S. J. (1974). Conjugal transfer of plasmid borne multiple antibiotic resistance in Streptococcus faecalis var. zymogenes. *J. Bacteriol.* 117. doi: 10.1128/jb.117.2.360-372.1974.

Law, J., Buist, G., Haandrikman, A., Kok, J., Venema, G., and Leenhouts, K. (1995). A system to generate chromosomal mutations in Lactococcus lactis which allows fast analysis of targeted genes. *J. Bacteriol.* 177. doi: 10.1128/jb.177.24.7011-7018.1995.

Marelli, B., and Magni, C. (2010). A simple expression system for Lactococcus lactis and Enterococcus faecalis. *World J. Microbiol. Biotechnol.* doi: 10.1007/s11274-009-0262-5.

Quintana, I. M., Gibhardt, J., Turdiev, A., Hammer, E., Commichau, F. M., Lee, V. T., et al. (2019). The KupA and KupB Proteins of Lactococcus lactis IL1403 Are Novel c-di-AMP Receptor Proteins Responsible for Potassium Uptake. *J. Bacteriol.* 201, 1–13. doi: 10.1128/JB.00028-19.

Schirmer, F., Ehrt, S., and Hillen, W. (1997). Expression, inducer spectrum, domain structure, and function of MopR, the regulator of phenol degradation in Acinetobacter calcoaceticus NCIB8250. *J. Bacteriol.* 179. doi: 10.1128/jb.179.4.1329-1336.1997.

Schlosser, A., Meldorf, M., Stumpe, S., Bakker, E. P., and Epstein, W. (1995). TrkH and its homolog, TrkG, determine the specificity and kinetics of cation transport by the Trk system of Escherichia coli. *J. Bacteriol.* 177. doi: 10.1128/jb.177.7.1908-1910.1995.

Stumpe, S., and Bakker, E. P. (1997). Requirement of a large K+-uptake capacity and of extracytoplasmic protease activity for protamine resistance of Escherichia coli. *Arch. Microbiol.* 167. doi: 10.1007/s002030050425.
