# Supplementary material for: Redundant potassium transporter systems guarantee the survival of Enterococcus faecalis under stress conditions
Source: Front Microbiol. 2023 Feb 8;14:1117684. doi: 10.3389/fmicb.2023.1117684 (PMC9945522; doi:10.3389/fmicb.2023.1117684)
Supplement: Supplementary file 2 [file Data_Sheet_1.PDF]

Redundant potassium transporter systems guarantee the survival of Enterococcus faecalis under stress conditions

Giuliana Acciarri, Fernán Oscar Gizzi, Mariano Alberto Torres Manno, Jörg Stülke, Martín Espariz, Victor Sebastian Blancato, Christian Magni

Experimental conditions for the supplementary material (Figures S1, S2, S3 and S4)

Growth of E. faecalis strains under stress conditions.

In order to compare the growth curves of wild-type and the different potassium transporter mutant strains, *E. faecalis* was cultivated in microplates at 37°C. For this, overnight cultures grown in LBG medium were used to inoculate fresh LBG medium adjusted to different initial pH values, as indicated. The inoculums were diluted to an initial OD_{600} of 0.1. After the cell culture had reached the exponential growth phase, the cells were harvested and washed twice with fresh medium. These samples were then diluted to an initial OD_{600} of 0.1 in LBG or mLBG medium at different initial pH, and supplemented with 10 mM KCl when indicated. The OD_{600} was registered every 20 minutes in a PowerWave™ XS Microplate reader.

For osmotolerance assays, cells were cultured as described above except with the addition of either NaCl or sorbitol, and KCl when indicated, at the specified concentrations. The growth rates were calculated and plotted against the NaCl concentrations, which ranged between 0 – 8%.

Infection and survival experiments in Galleria mellonella

Infection and survival experiments in *G. mellonella* were carried out as described (Terán et al., 2022).

Growth in urine or blood.

Growth in urine or blood was analyzed as described previously (Martino et al., 2018).
