# Supplementary figures and images for: Redundant potassium transporter systems guarantee the survival of Enterococcus faecalis under stress conditions
Source: Front Microbiol. 2023 Feb 8;14:1117684. doi: 10.3389/fmicb.2023.1117684 (PMC9945522; doi:10.3389/fmicb.2023.1117684)

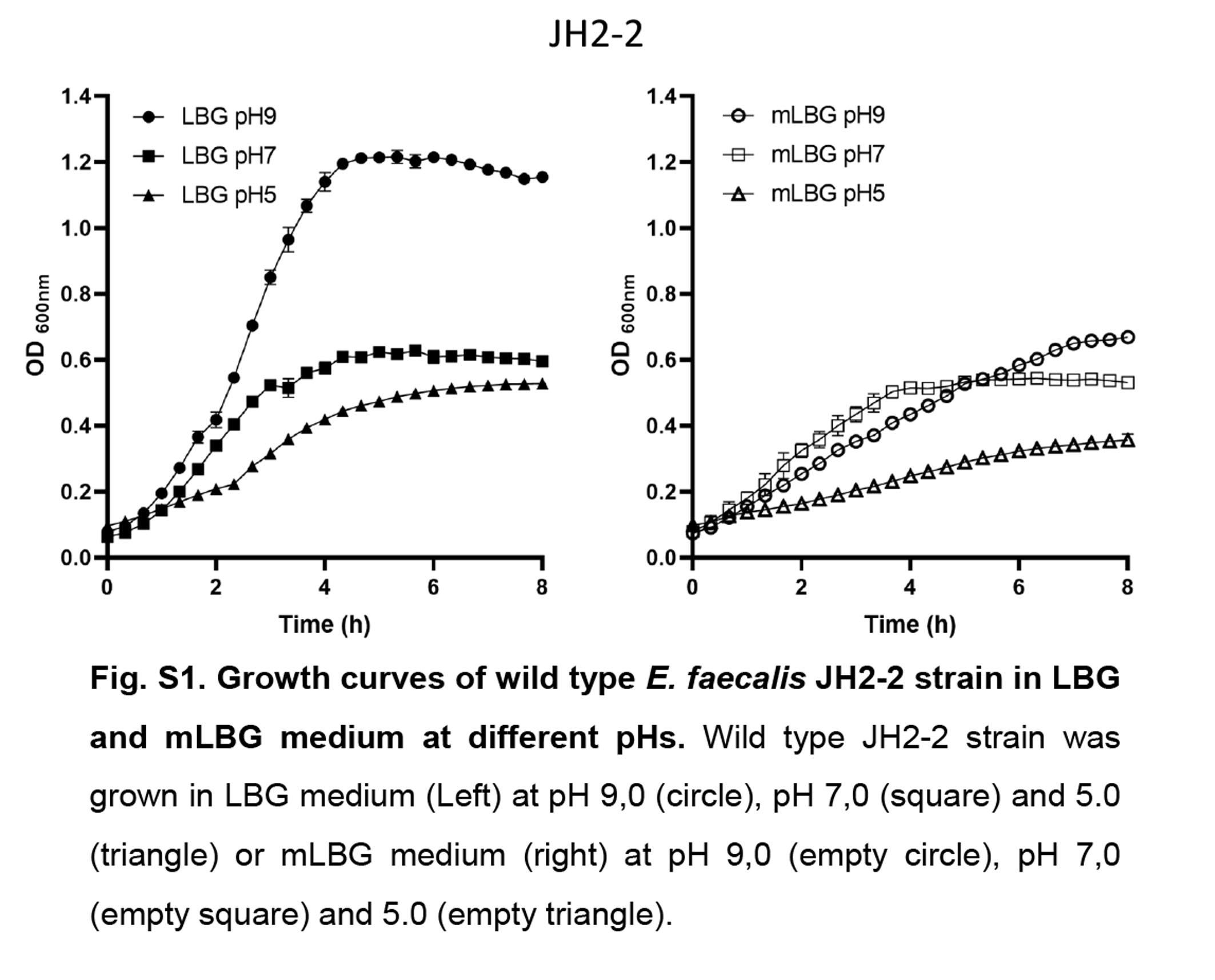

Supplement: Supplementary file 3 [file Image_1.JPEG]

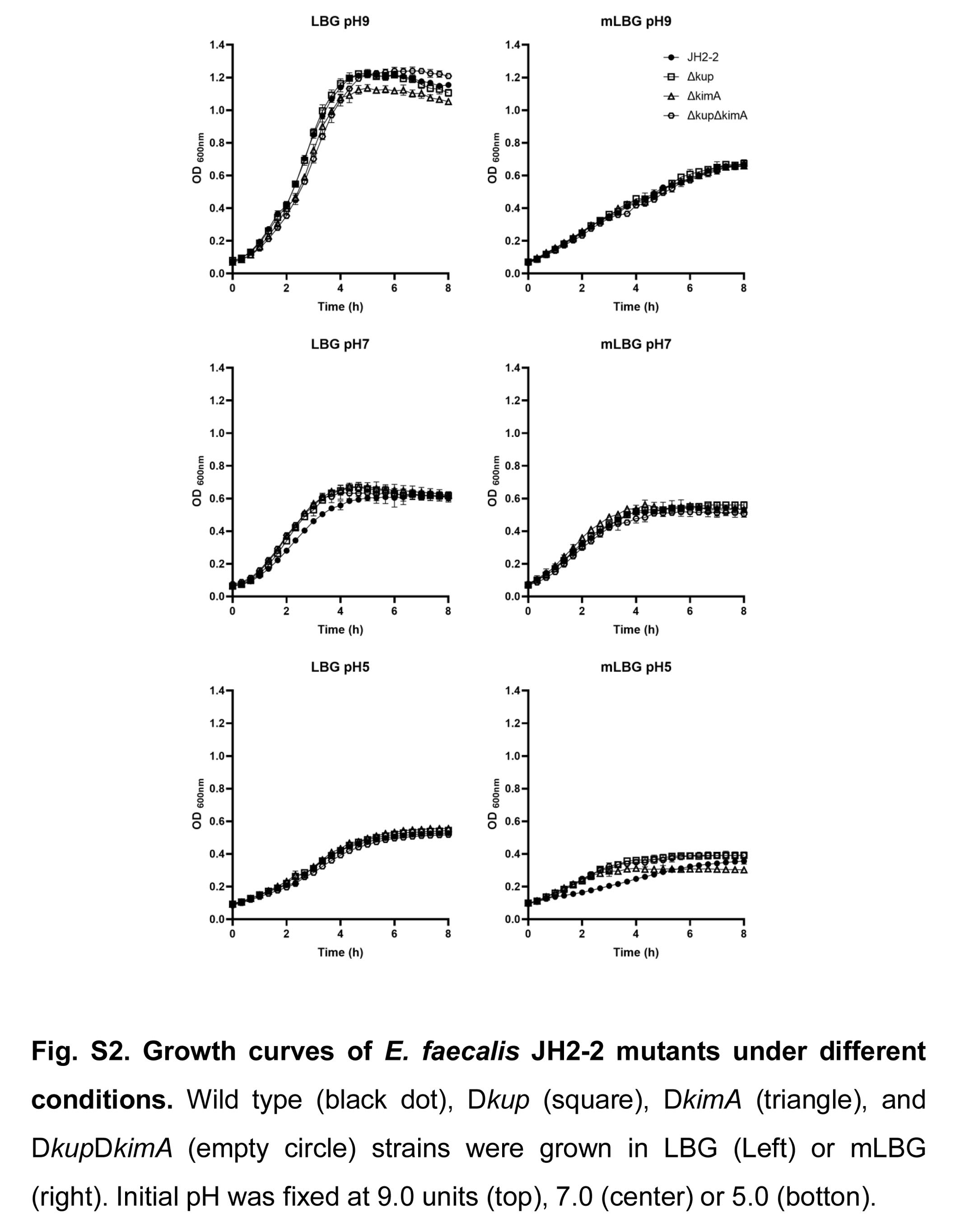

Supplement: Supplementary file 4 [file Image_2.JPEG]

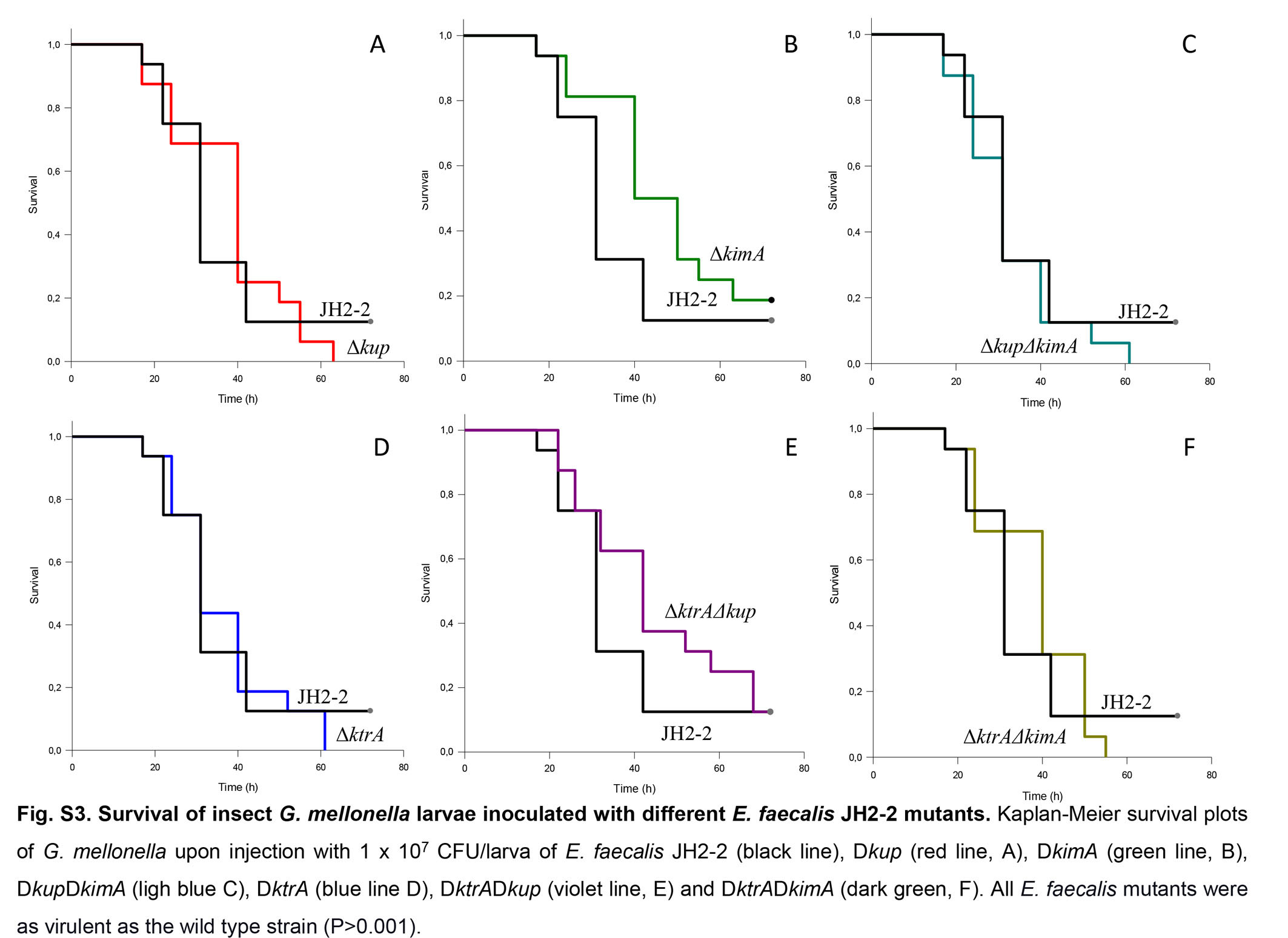

Supplement: Supplementary file 5 [file Image_3.JPEG]

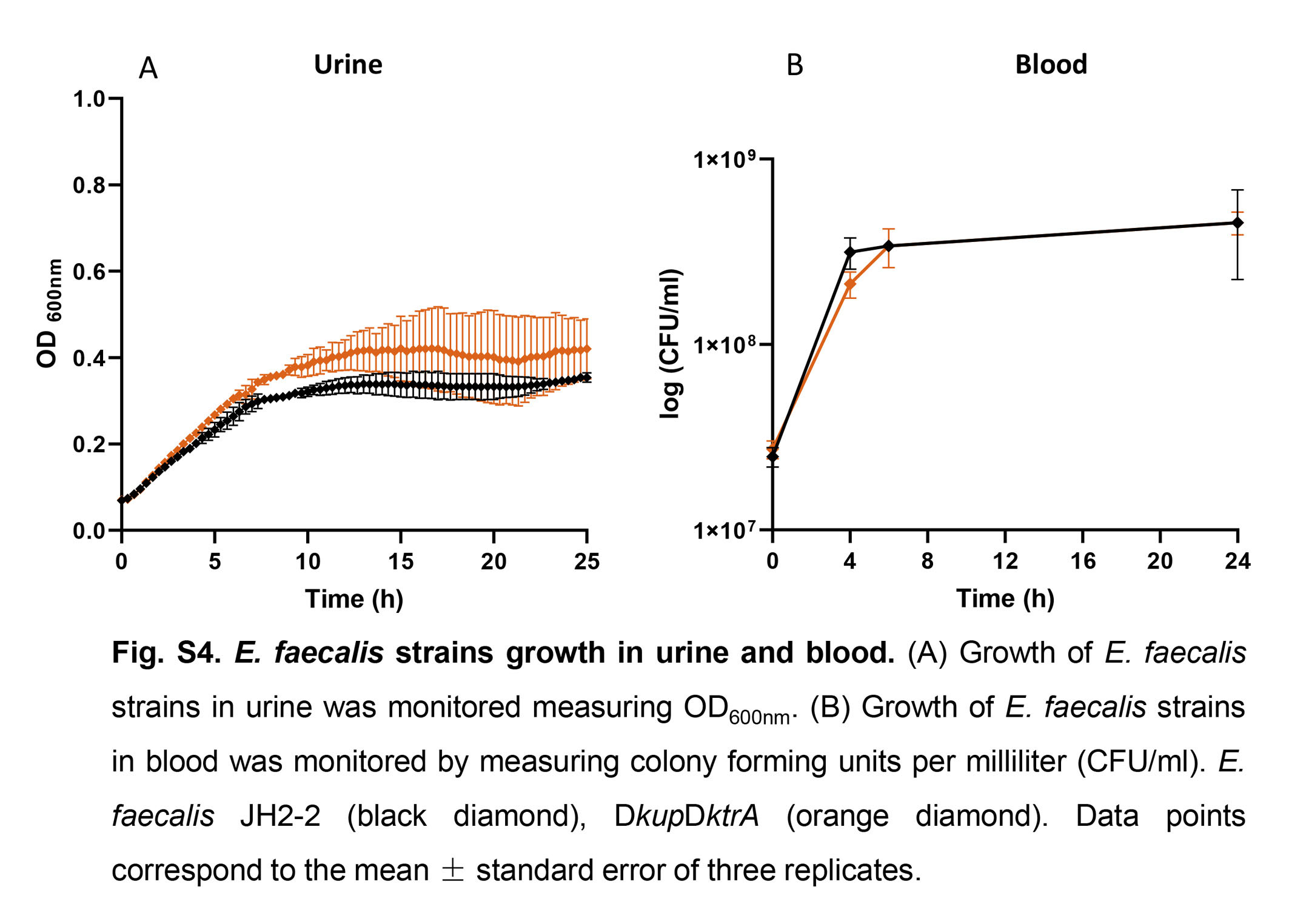

Supplement: Supplementary file 6 [file Image_4.JPEG]
